# Genotypic capacity of post-anthesis stem reserve mobilization in wheat for yield sustainability under drought and heat stress in the subtropical region

**DOI:** 10.3389/fgene.2023.1180941

**Published:** 2023-06-20

**Authors:** S. Gurumurthy, A. Arora, Hari Krishna, V. Chinnusamy, K. K. Hazra

**Affiliations:** ^1^ Division of Plant Physiology, ICAR–Indian Agricultural Research Institute, New Delhi, India; ^2^ School of Water Stress Management, ICAR–National Institute of Abiotic Stress Management, Baramati, Maharashtra, India; ^3^ Division of Genetics, ICAR–Indian Agricultural Research Institute, New Delhi, India; ^4^ Crop Production Division, ICAR–Indian Institute of Pulses Research, Kanpur, Uttar Pradesh, India

**Keywords:** stem reserve mobilization, drought stress, terminal heat stress, defoliation, grain yield, wheat

## Abstract

Wheat productivity is severely affected by drought and heat stress conditions worldwide. Currently, stem reserve mobilization (SRM) is receiving increased attention as a trait that can sustain wheat yields under adverse environments. However, the significance of SRM in sustaining wheat yields under drought and heat stress conditions remains uncertain in the tropical climate of Indo-Gangetic Plain region. Therefore, this study aimed to investigate genotypic variations in SRM in wheat and their influence on yield sustainability under drought and heat stress environments. The experiment was designed in an alpha-lattice layout, accommodating 43 genotypes under four simulated environments [timely sown and well irrigated (non-stress); timely sown and water-deficit/drought stress; late-sown and well-irrigated crop facing terminally high temperature; and late-sown and water-deficit stress (both water-deficit and heat stress)]. The water-deficit stress significantly increased SRM (16%–68%, *p* < 0.01) compared to the non-stress environment, while the heat stress conditions reduced SRM (12%–18%). Both SRM and stem reserve mobilization efficiency exhibited positive correlations with grain weight (grain weight spike^−1^) under all three different stress treatments (*p* < 0.05). Strong positive correlations between stem weight (at 12 days after anthesis) and grain weight were observed across the environments (*p* < 0.001); however, a significant positive correlation between stem weight and SRM was observed only with stress treatments. Results revealed that the SRM trait could effectively alleviate the impacts of water-deficit stress on yields. However, the SRM-mediated yield protection was uncertain under heat stress and combined water-deficit and heat stress treatments, possibly due to sink inefficiencies caused by high temperature during the reproductive period. Defoliated plants exhibited higher SRM than non-defoliated plants, with the highest increment observed in the non-stress treatment compared to all the stress treatments. Results revealed that wider genetic variability exists for the SRM trait, which could be used to improve wheat yield under drought stress conditions.

## Introduction

Wheat is the most widely cultivated food crop globally, contributing to ∼ approximately 20% of the daily dietary energy total calories and protein for 4.5 billion people (Gooding and Shewry, 2022). The Indo-Gangetic Plain (IGP) is one of the main wheat-producing regions in India and worldwide (Daloz et al., 2021). India is the second largest wheat producer globally, contributing 13% of the wheat supply and exporting 0.2 million tons annually (Zaveri, and Lobell, 2019). In the IGP region, wheat productivity is affected by drought and terminal heat stresses (Zaveri, and Lobell, 2019; Chowdhury et al., 2020). In tropical climates, heat stress at the terminal growth stage causes significant yield losses (21%–30%) in wheat under late-sown conditions (Wang H. et al., 2016). Similarly, midseason or terminal water-deficit stress or drought conditions threaten wheat productivity worldwide (Farooq et al., 2015). The primary yield-limiting factors under drought and heat stress environments include source limitations due to reduced photosynthate assimilation, oxidative damage to cells, membrane disruption, forced maturity, sink inefficiencies, and poor grain filling (Chowdhury et al., 2019). Consequently, there is an urgent need to develop stress-tolerant/adaptive cultivars by deploying stress-inducive functional traits to sustain wheat yields.

Stem-assimilate reserves serve as a carbon source for developing grains in wheat, and they are prominent when photosynthate assimilation during grain filling is affected by abiotic stress factors (Shirdelmoghanloo et al., 2016). The scale of stem reserve mobilization (SRM) in wheat varies from 10% to 20% of the total grain weight under non-stressed conditions, and it can increase up to 30%–50% under drought and heat stress conditions (Shearman et al., 2005; Ehdaie et al., 2008). Therefore, deploying the genetic potential of SRM could be one way of improving crops under adverse environments of tropical agro-regions, where wheat growth is more source-limited than in temperate regions (Cruz-Aguado et al., 1999). Our current understanding of the impact of traits such as stem-assimilate reserves, sink capacity and efficiencies, grain-filling duration, and senescence dynamics on SRM in wheat under different stress conditions in tropical regions is limited. Although the contribution of stem reserve assimilate toward grain development in wheat has been studied and reported by several researchers (Ehdaie et al., 2008; Zhu et al., 2009; Talukder et al., 2013; Khoshro et al., 2014), the comparative assessment of SRM under drought and heat stress (or combined drought and heat stress) on the same platform is presently lacking.

Plant functional traits, such as stem-assimilate reserve capacity, SRM, and stem reserve mobilization efficiency (SRE), have a significant impact on crop yield depending on genotypes and the intensity of stress (Ehdaie et al., 2006; Ruuska et al., 2006). To strategically deploy the SRM trait for crop improvement, it is necessary to have an improved understanding of its agronomic, physiological, and genetic basis under diverse stress conditions. Secondary functional traits, including SRM, membrane stability, photosynthetic rate, and grain weight stability, should be considered in wheat pre-breeding and breeding to alleviate abiotic stresses (Farooq et al., 2011). Thus, evaluating genotypic variations for SRM and SRE under stress conditions is crucial to identify the genotype(s)/breeding line(s) with higher levels of SRM-mediated stress-tolerant capacity and to comprehend the primary determinants of these processes.

Therefore, a field experiment was conducted in regards to the tropical climate of India (New Delhi) to assess the potential of SRM (stem reserve mobilization) in sustaining wheat yields under water-deficit and high-temperature stress conditions and to assess the genotypic variability for the trait. The sensitivity of traits of SRM and SRE under non-stress and stressful environments (water-deficit stress, heat stress, and combined water-deficit and heat stress) were evaluated using a panel of 43 wheat genotypes and their associations with grain yield and yield parameters, plant growth, and physiological attributes were determined. The major objectives of the study were to i) quantify the genotypic variations for the SRM trait and contribution of SRM toward grain development and sustaining wheat yield under drought and heat stress environments in tropics, ii) understand the physiological basis of SRM in wheat under drought and heat stress environments, iii) evaluate the interactive effect of genotype × environment on the SRM trait in wheat, and iv) quantify the scale of SRM under drought and heat stress environments in the absence of a primary photosynthesis organ during the grain-filling period (defoliation study).

## Materials and methods

### Site characteristics

The field experiment was carried out during the winter season of 2016–2017 and 2017–2018 at the ICAR–Indian Agricultural Research Institute (ICAR–IARI) research farm New Delhi, India (28°41^/^ N and 77°13^/^ E, 228 m above the sea level). The site has a sub-humid tropical climate, and weather conditions during the cropping period are shown in Supplementary Figure S1. The weather variables were recorded from a meteorological observatory located at the ICAR–IARI research farm. Maximum and minimum temperatures were recorded from the maximum and minimum temperature thermometers installed inside the Stevenson screen, while the evaporation rate and rainfall were recorded using a USWB Class-A pan evaporimeter and a rain gauge, respectively. The experimental soil belongs to the *Fluvisol* order (World Reference Base soil classification). The soil had pH 8.05 and organic carbon 4.3 g kg^–1^, and the available nitrogen, phosphorus, and potassium were 117.6, 7.4, and 132.4 mg kg^–1^, respectively.

### Treatment description and experimental design

**FIGURE 1:**
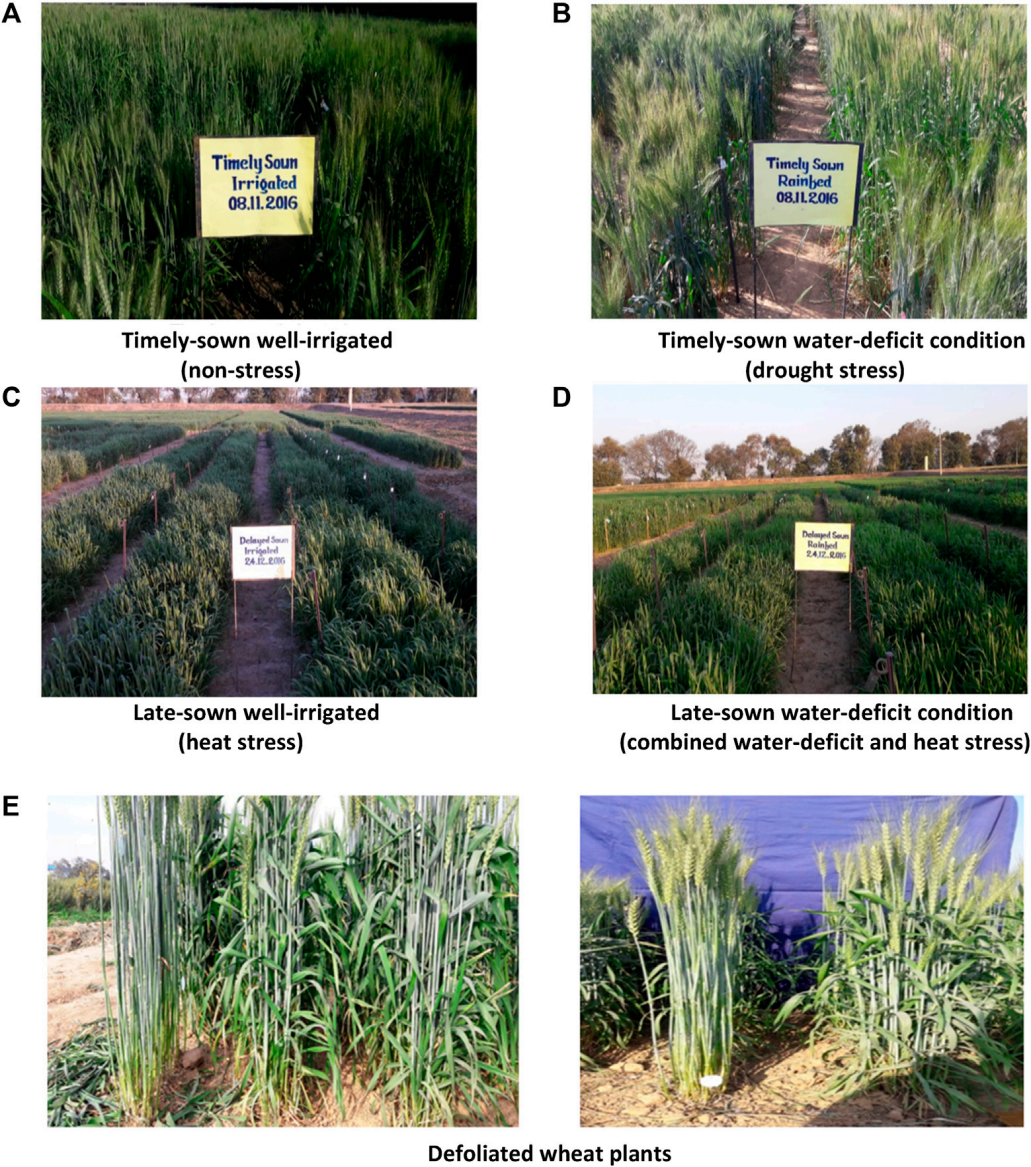
Field view of wheat crop under different environments **(A–D)** and defoliated wheat plants **(E)**.

In this study, a panel of 43 wheat genotypes with contrasting traits was selected (Supplementary Table 1). The genotypes were chosen based on their contrasting heat tolerance and susceptibility (6 + 6 genotypes), drought tolerance and susceptibility (7 + 7 genotypes), plant height (four tall and five dwarf genotypes), maturity duration (four early- and four late-maturing genotypes), RILs (9 + 2 parents), current best standard genotypes or best-adapted varieties (six genotypes), and popular ruling varieties from the last century (10 genotypes). The list of genotypes under different groups is shown in the Supplementary Material. The genotypes were evaluated under four different growing conditions: timely sown well-irrigated condition (non-stress), timely sown deficit irrigation condition (water-deficit stress), late-sown and well-irrigated condition (terminal heat stress), and late-sown deficit irrigation condition (combined terminal heat and drought stress) (Figure 1). The experiment was laid out following an alpha-lattice design with two replications (Patterson and Williams, 1976). Each genotype was sown manually in a gross plot size of 4 m × 1 m with an inter-row spacing of 23.0 cm. The timely sown and late-sown crops were sown on 8 November and 23 December, respectively, in both years. The late-sown crops were sown 45 days later than the timely sown crops to expose them to a terminal heat stress environment. Water-deficit stress was imposed by scheduling/withholding irrigation. In treatments with well-irrigated conditions, irrigation was applied at critical growth stages, including crown root initiation, active tillering, jointing, flowering, and dough stages (8 ha–cm each irrigation). In treatments with deficit-water conditions, life-saving irrigation (4 ha–cm each irrigation) was applied at crown root initiation, active tillering, jointing, and flowering stages, but no irrigation was applied at the dough stage.

In the year 2016–2017, from each plot of the experimental field, a complete row of 4 m length was defoliated by cutting off all the leaf blades at 12 days after anthesis (Figure 1). The objective of the study was to assess the changes in SRM in defoliated plants as compared to the non-defoliated (intact plants). This study was a part of the main experiment conducted during the year 2016–2017, and therefore, all the crop-growing environment treatments, panel of genotypes, and crop management practices were similar to that of the main experiment as described previously.

### Measurements

#### Soil moisture content

Soil moisture content (w/w) was determined by the gravimetric method. Soil samples from each experimental unit were collected using a post-hole auger, and soils were placed in aluminum boxes with secure lids. The samples were weighed immediately and then oven-dried at 105°C for 72 h, and soil moisture content (%) was calculated using the following formula:

$$Soil\;moisture\;\left(\%\right)=\frac{Weight\;of\;moist\;soil\;\left(g\right)-Weight\;of\;oven\;dry\;soil\;\left(g\right)}{Weight\;of\;oven\;dry\;soil\;\left(g\right)}\times 100.$$ (1)

#### Crop phenology and growth attributes

Crop phenological events such as days to anthesis and days to maturity were recorded for each genotype/breeding line. The anthesis stage was determined visually for each genotype when anther extrusion occurs in 50% of the ears. The days taken from the sowing to anthesis stage was denoted as days to anthesis. The maturity (physiological maturity) of each genotype was determined both by visual (when all the leaves and spike turned to a complete yellowish color) and sensor-based observations (SPAD 502 chlorophyll meter reading and normalized differential vegetation index were shown in the range of <5 and <0.20, respectively) (reference). SPAD meter reading and normalized differential vegetation index were measured with the instrument SPAD 502 m (Konica–Minolta, Japan) and handheld Ntech “Greenseeker” (field portable NDVI sensor), respectively. The days taken from anthesis to physiological maturity was calculated and denoted as the reproductive period (days).

Five primary tillers (main stem) of each genotype were collected 12 days after anthesis. Then, the stem, leaf, and ear parts of the tillers were separated. The length of every single stem was recorded, and the stem part was oven-dried (65°C for 72 h) and dry-weight was recorded. The stem-specific weight (g cm^–1^) was calculated as the ratio of stem dry weight (g) to its length (cm). Likewise, at the physiological maturity, again five primary tillers were sampled in each genotype and stem weight and length were recorded. The dry-weight of the grain in a spike from the five selected primary tillers was recorded at the time of crop maturity. The ratio of grain weight per spike to the total weight of the main tiller (including spike) was calculated to determine the harvest index (HI) and it is expressed as percentage.

For the defoliation experiment (2016–2017), similar observations (main stem weight) at 12 days after anthesis and physiological maturity were recorded from defoliated plants. Grain weight per spike was recorded and grain weight percentage at physiological maturity was calculated like non-defoliated plants mentioned previously.

### Temperature intensity and growing degree day

The average ambient maximum temperature (°C) during flowering (*i.e.*, at anthesis ±5 days) was calculated for each genotype to determine the temperature intensity during the flowering stage. Likewise, for each genotype, the average ambient maximum temperature (°C) during the reproductive period, *i.e.*, anthesis to physiological maturity was calculated using the daily maximum temperature data. The mean ambient maximum temperature (°C) during the anthesis and reproductive period of an environment represents the average of all genotypes. Growing degree days from anthesis to physiological maturity were calculated using the following formula (Santiveri et al., 2002):

$$Growing\;degree\;day\;\left(degree\;C\right)=\sum \left(\frac{Tmax+Tmin}{2}-base\;temperature\right),$$ (2)

where *T*max and *T*min represent the maximum and minimum temperatures, respectively. Here, the base–temperature (0°C) defines the minimum threshold temperature below which the crop development is ceased.

### Calculation of stem reserve mobilization and stem reserve mobilization efficiency

Ten uniform plants of each genotype (those plants were selected randomly where flowers appear on the same days) within a plot were tagged for sampling. Five tagged plants were sampled at 12 days after anthesis, and the remaining five plants were collected at crop maturity. According to reports, the maximum stem weight and carbohydrate concentration occur at 10–14 days after anthesis, and after that, there is a rapid decline in carbohydrate concentration in stems (Lopatecki et al., 1962; Ford et al., 1979). Stem reserve mobilization represents the amount of dry matter mobilized from stem to grain. The SRM value was calculated by the difference of stem dry weight at 12 days after anthesis and maturity (Eq. 3). Stem reserve mobilization efficiency was calculated as the proportional contribution percentage (%) of mobilized stem reserve to stem weight at 12 days after anthesis (Ehdaie et al., 2006) (Eq. 4).

$$SRM\;\left(g\right)=\left[Stem\;weight\;at\;twelve\;days\;after\;anthesis\;\left(g\right)-Stem\;weight\;at\;maturity\left(g\right)\right],$$ (3)

$$SRE\;\left(\%\right)=\frac{Stem\;weight\;at\;twelve\;days\;after\;anthesis\;\left(g\right)-Stem\;weight\;at\;maturity\left(g\right)}{Stem\;weight\;at\;twelve\;days\;after\;anthesis\;\left(g\right)}\times 100.$$ (4)

In Eq. 3, the contribution of SRM to grain weight was calculated as the grain weight percentage by using the following formula:

$$Grain\;weight\;percentage\;\left(\%\right)=\frac{Stem\;reserve\;mobilation\;\left(g\right)}{Weight\;of\;grains\;per\;spike\;\left(g\right)}\times 100.$$ (5)

For the defoliation study (2016–2017), SRM and SRE were estimated following the same procedure as mentioned previously.

### Grain yield estimation

A net area of 1.35 m^2^ (3 m row length × 0.45 m) was harvested from each genotype plot to estimate the grain yield. A sub-sample from the harvested grains was analyzed for moisture content (%, w/w), and the grain yield was adjusted to 12% moisture content (w/w) and expressed as kg ha^–1^. The grain yield loss of each genotype under different stress conditions was calculated by the difference of yield of timely sown well-irrigated condition (non-stress) treatment and stress treatments (drought stress, heat stress, and combined drought and heat stress).

### Statistical analysis

The pooled analysis of variance was analyzed considering the year (*n* = 2), environments (*n* = 4), and genotypes (*n* = 43) as the main, sub, and sub–sub factors, respectively. The META–R program (multi environment trail analysis with R for Windows) was used for computing by fitting mixed and fixed linear models from the alpha-lattice experimental design (Alvarado et al., 2020). For the alpha-lattice design the model is explained as

$$Y_{ijkl}=\mu +{Loc}_{i}+{Rep}_{j}\;\left({Loc}_{i}\right)+{Block}_{k}\;\left({Loc}_{i}{Rep}_{j}\right)+{Gen}_{l}+{Loc}_{i}\;{\times Gen}_{l}+Cov+\varepsilon_{ijkl},$$ (5a)

where *Y*_*ijkl*_ is the trait of interest, *μ* is the overall mean effect, *Loc*_*i*_ is effects of the *ith* environment, *Loc*_*i*_ *× Gen*_*k*_ are the effects environment × genotype (G × E) interaction, *Rep*_*j*_ is the effect of the *jth* replicate, *Block*_*k*_ the effect of the *kth* incomplete block, *Gen*_*l*_ is the effect of the *lth* genotype, Cov is the effect of the covariate, and *ε*_*ijkl*_ is the effect of the error associated with the *ith* environment, *jth* replication, *kth* incomplete block, and *lth* genotype.

The mean comparison of the different environments was performed following Tukey’s test using SPSS statistical software (version 20.0.6). The linear regression analysis was performed using the R statistical package (version 3.5.2). Box plots of SRM and SRE parameters for non-defoliated crops were graphically presented using PAST statistical software (version 3.14). The mean comparison of plant traits and SRM of defoliated and non-defoliated plants was conducted by a paired *t*-test. Genotype + genotype-by-environment (GGE) biplots were developed using the R studio platform using the ‘‘GGEbiplotGUI’’ package (Yan and Tinker, 2006). The GGEbiplots were constructed by plotting the first two principal components derived by subjecting the mean values to singular-value decomposition (Yan and Tinker, 2006). To display the mean performance and stability of a genotype, the biplots were framed with the mean vs. stability function by adopting no scaling (scale = 0), tester–centred G + GE (centring = 2) with genotype focused (row metric preserving) singular-value partitioning (SVP = 1) (Parihar et al., 2018), (Eqs 5, 5a).

## Results

### Weather condition and soil moisture

**FIGURE 2:**
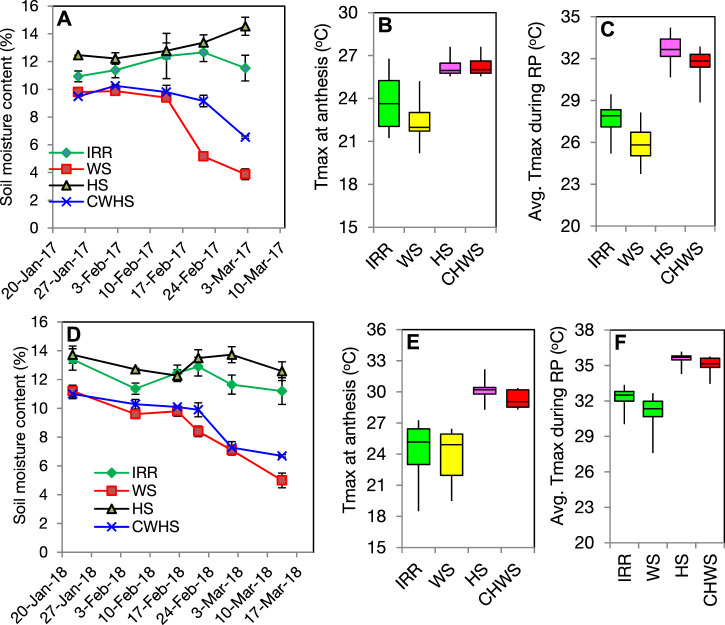
Temporal dynamics of soil moisture (w/w) during crop season of 2016–2017 **(A)** and 2017–2018 **(D)**. Box plot presentation of ambient maximum temperature (Tmax, oC) at anthesis stage of wheat as influenced by different crop growing environments in the year 2016–2017 **(B)** and 2017–2018 **(E)**. Box plot presentation of ambient maximum temperature (Tmax, oC) during the reproductive period (anthesis to maturity) of wheat crop as influenced by different crop growing environments in the year 2016-2017 **(C)** and 2017–2018 **(F)**. IRR, timely sown well-irrigated (non-stress); WS, water-deficit stress; HS, heat stress; and CWHS, combined water-deficit and heat stress.

In the cropping year (2016–2017), soil moisture content during the grain-filling period (anthesis to maturity) was recorded to be the highest in the heat-stress treatment (13.1%), followed by timely sown irrigated (11.4%), combined water and heat stress (8.8%) and water-deficit stress (6.3%) treatments (Figure 2). Likewise, in the year 2017–2018, the soil moisture during the grain-filling period followed the same treatment order, *i.e.*, heat stress (12.9%) ≥ timely sown irrigated (12.3%) > combined water and heat stress (8.3%) > water-deficit stress (5.8%). Soil moisture gradient within the treatments was prominent after the anthesis stage and recorded as the highest at the end of the crop season (or maturity). In both cropping years, the ambient maximum temperature during flowering was higher in the heat stress and combined water-deficit and heat stress treatments (Figure 2), while the mean maximum temperatures during the reproductive period were significantly higher in the heat stress and combined water and heat stress treatments over the water-deficit stress, and non-stress (timely sown irrigated) treatments.

### Impact of drought and terminal heat stress on wheat crop

Water-deficit stress had reduced plant height (7%–16%, *p* < 0.01), stem weight (anthesis) (11%–12%, *p* < 0.01), reproductive period (8%, *p* < 0.05), and grain weight spike^−1^ (12%–16%, *p* < 0.01) as compared to the non-stress environment (Table 1). The heat-stress treatment reduced the plant height, stem weight, stem-specific weight, reproductive period, and grain weight spike^−1^ by 8%–27%, 28%–42%, 21%–22%, 29%–32%, and 20%–42%, respectively (*p* < 0.05), compared to the non-stress environment. Likewise, the corresponding reductions with the combined water-deficit and heat stress environment with reference to the non-stress environment were 18%–30%, 30%–48%, 13%–25%, 32%–33%, and 38%–39% (*p* < 0.01). Compared to the non-stress environment, the reductions in grain yield with water-deficit stress, heat stress, and combined water-deficit and heat stress environments were 26%–28%, 56%–61%, and 73%–79%, respectively (*p* < 0.01). The best linear unbiased estimator (BLUE) model explained that the genotype × environment interaction was highly significant on stem weight, grain weight spike^−1^, and grain yield (*p* < 0.001) (Table 2).

**TABLE 1:** Growth and yield attributing parameters of wheat as influenced by non-stressed and stressed environments.

Year	Parameter	Environment			
		Irrigated (non-stress)	Water-deficit stress	Heat stress	Combined water-deficit and heat stress
2016–2017	Plant height (cm)	93.8 ± 3.5^a^	87.0 ± 4.1^b^	86.3 ± 3.15^b^	76.6 ± 2.68^c^
	Single stem weight at 12 DAA (g)	2.10 ± 0.12^a^	1.87 ± 0.11^b^	1.52 ± 0.08^c^	1.48 ± 0.07^c^
	Single stem weight at maturity (g)	1.80 ± 0.11^a^	1.46 ± 0.07^b^	1.27 ± 0.07^c^	1.15 ± 0.06^c^
	Stem-specific weight (g cm^−1^)	22.03 ± 1.01^a^	22.48 ± 0.99^a^	17.39 ± 0.73^c^	19.12 ± 0.80^b^
	Reproductive period (day)	42.2 ± 1.7^a^	38.9 ± 1.1^b^	29.8 ± 0.9^c^	28.5 ± 0.7^c^
	Grain weight per spike (g)	2.68 ± 0.11^a^	2.25 ± 0.09^b^	1.55 ± 0.09^c^	1.66 ± 0.07^c^
	Grain weight percentage (%)	11.5 ± 2.7^c^	22.4 ± 2.4^a^	15.8 ± 2.3^bc^	20.2 ± 2.3^ab^
	Harvest index (%)	0.38 ± 0.02^a^	0.37 ± 0.02^a^	0.23 ± 0.02^c^	0.32 ± 0.01^b^
	Growing degree days (°C-day)^#^	843.4 ± 28.1^a^	739.8 ± 18.1^bc^	763.1 ± 14.8^b^	705.8 ± 16.8^c^
2017–2018	Plant height (cm)	84.8 ± 2.6^a^	70.9 ± 1.8^b^	61.9 ± 3.7^c^	59.7 ± 4.0^c^
	Single stem weight at 12 DAA (g)	2.30 ± 0.09^a^	2.03 ± 0.08^b^	1.32 ± 0.06^c^	1.19 ± 0.05^d^
	Single stem weight at maturity (g)	1.91 ± 0.09^a^	1.57 ± 0.06^b^	0.95 ± 0.04^c^	0.78 ± 0.03^d^
	Stem-specific weight (g cm^−1^)	26.11 ± 0.91^a^	26.48 ± 0.73^a^	20.39 ± 0.71^b^	19.72 ± 0.78^b^
	Reproductive period (day)	43.14 ± 1.62^a^	39.00 ± 1.03^b^	30.11 ± 0.89^c^	28.73 ± 0.65^c^
	Grain weight per spike (g)	2.64 ± 0.09^a^	2.32 ± 0.09^b^	2.12 ± 0.07^c^	1.48 ± 0.05^d^
	Grain weight percentage (%)	15.39 ± 2.46^c^	19.63 ± 1.98^b^	17.49 ± 1.26^bc^	27.70 ± 1.68^a^
	Harvest index (%)	0.37 ± 0.02^a^	0.36 ± 0.02^a^	0.20 ± 0.03^d^	0.29 ± 0.02^c^
	Growing degree days (°C-day)	889.1 ± 22.1^a^	769.8 ± 14.4^b^	768.1 ± 16.9^b^	713.2 ± 11.20^c^

*DAA*, days after anthesis; the values represent the mean ±95% confidence interval.

# Growing degree-days value represents to the cumulative degree days during reproductive period (anthesis to physiological maturity);

^a^ Different uppercase letters within the row values are the significant difference at *p* = 0.05 according to Tukey’s test.

^b^ Different uppercase letters within the row values are the significant difference at *p* = 0.05 according to Tukey’s test.

^c^ Different uppercase letters within the row values are the significant difference at *p* = 0.05 according to Tukey’s test.

^d^ Different uppercase letters within the row values are the significant difference at *p* = 0.05 according to Tukey’s test.

**TABLE 2:** Best linear unbiased estimators of parameters stem reserve mobilization, stem reserve mobilization efficiency, stem weight, grain weight spike^−1^, and grain yield based on 2-year experimental data.

Environment	Statistic	SRM	SRE	Stem weight	Grain weight spike^−1^	Grain yield
OVERALL	Heritability	0.497215	0.564039	0.73249	0.568307	0.363365
	Genotype variance	0.002719	6.867062	0.020052	0.010692	0.007774
	Genotype × local variance	0.008539	7.98844	0.038534	0.013855	0.159308
	Residual variance	0.026915	68.94685	0.0401	0.102242	0.318399
	Grand mean	0.376819	22.19486	1.738171	2.083643	2.512193
	Least significant difference	0.103215	4.848527	0.20395	0.189365	0.225201
	Coefficient of variation	43.53793	37.41148	11.52075	15.34589	22.46121
	Replicates (*n*)	2	2	2	2	2
	Environments (*n*)	8	8	8	8	8
	Genotype significance (*p*-value)	0.00124	7.94 × 10^−05^	2.69 × 10^−11^	6.28 × 10^−05^	0.0339698
	Genotype × environment significance (*p*-value)	1.24 × 10^−05^	0.067198	1.53 × 10^−21^	0.032653	2.61 × 10^−06^

The grain weight spike^−1^ in defoliated plants was lower than in the non-defoliated plants (paired *t*-test, *p* < 0.05), and the highest reduction was recorded in the non-stress environment (25%) followed by water-deficit (19%) and heat stress (13%) and the lowest in the combined water-deficit and heat stress environment (7%) (*p* < 0.05) (Table 3).

**TABLE 3:** Effect of different crop-growing environments on stem and grain attributes of defoliated wheat plants.

Parameter	Environment			
	Irrigated (non-stress)	Water-deficit stress	Heat stress	Combined water-deficit and heat stress
Single stem weight at 12 DAA (g)	2.10 ± 0.12^a^	1.87 ± 0.11^b^	1.52 ± 0.08^c^	1.48 ± 0.07^c^
Single stem weight at maturity (g)	1.63 ± 0.10^a^	1.43 ± 0.08^b^	1.25 ± 0.07^c^	1.14 ± 0.06^c^
Grain weight per spike (g)	1.71 ± 0.04^a^	1.55 ± 0.04^b^	1.16 ± 0.04^d^	1.32 ± 0.03^c^
Grain weight percentage (%)	28.6 ± 8.1^ab^	35.7 ± 8.3^a^	23.6 ± 5.4^b^	27.1 ± 6.5^b^

^a^ Different uppercase letters within the row values are the significant difference at *p* = 0.05 according to Tukey’s test. *DAA*, days after anthesis.

^b^ Different uppercase letters within the row values are the significant difference at *p* = 0.05 according to Tukey’s test. *DAA*, days after anthesis.

^c^ Different uppercase letters within the row values are the significant difference at *p* = 0.05 according to Tukey’s test. *DAA*, days after anthesis.

^d^ Different uppercase letters within the row values are the significant difference at *p* = 0.05 according to Tukey’s test. *DAA*, days after anthesis.

### Stem reserve mobilization and its relation with yield parameters

**FIGURE 3:**
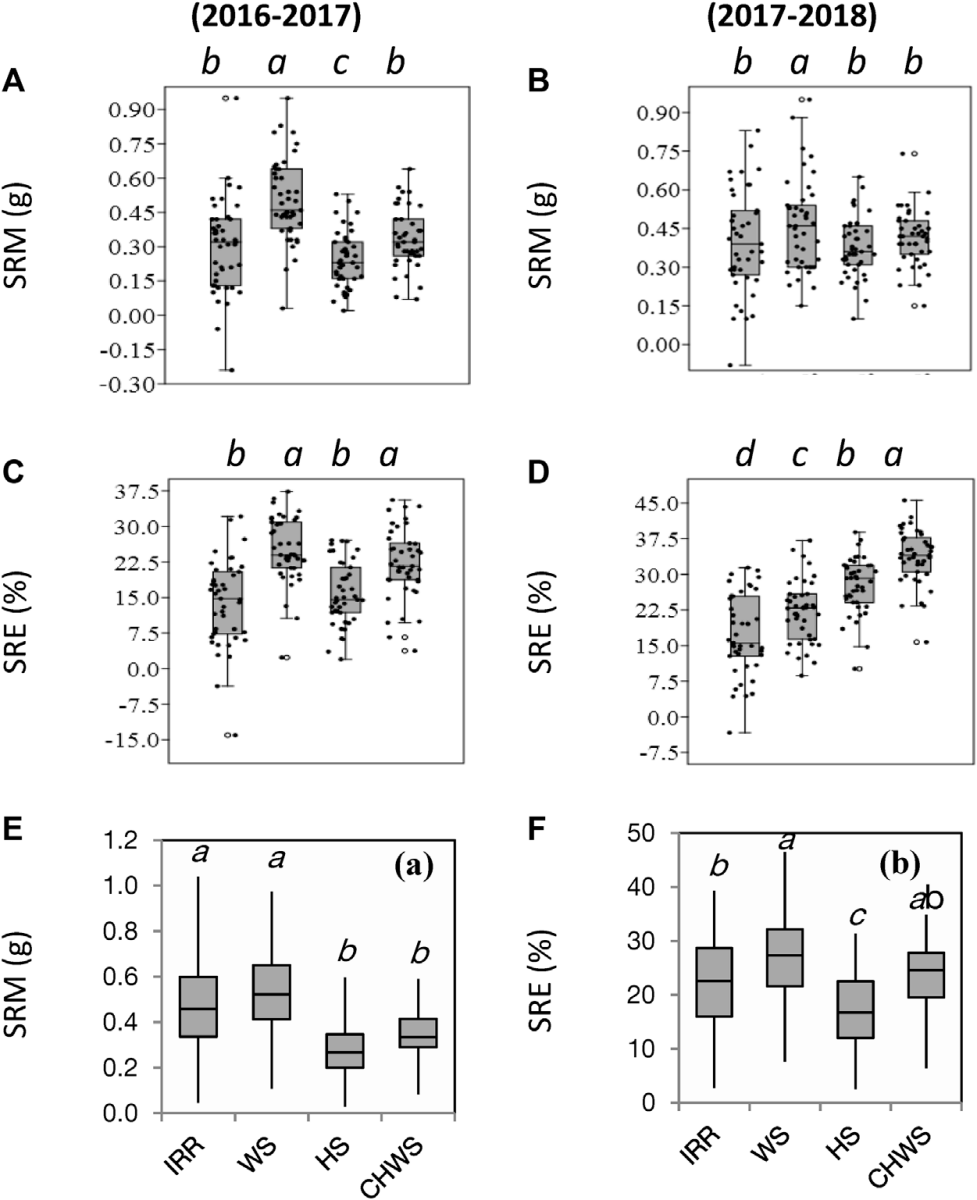
Box plot presentation of parameters stem reserve mobilization (SRM) **(A, C)** and stem reserve mobilization efficiency (SRE) **(B, D)** as influenced by different crop growing environments (2016–2017 and 2017–2018). Box plot presentation of stem reserve mobilization (SRM) **(E)** and stem reserve efficiency (SRE) **(F)** of defoliated wheat plants as influenced by different crop growing environments in the year 2016–2017 (coloured graphs). IRR, irrigated (non-stress); WS, water-deficit stress; HS, heat stress; and CWHS, combined water-deficit and heat stress. Each data point represents a single genotype. a–d, different lowercase letters corresponding to the treatments represent significantly different at *p < 0.05* as per Tukey’s test.

Water-deficit stress increased SRM by 68% and 16% over the non-stress environment (timely sown well irrigated) in the year 2016–2017 and 2017–2018, respectively (*p* < 0.05) (Figure 3). The heat stress treatment reduced SRM (12%–18%) compared to the non-stress environment, but the reduction was significant only in the year 2016–2017 (*p* < 0.05). SRM values were comparable within the non-stress, heat stress, and combined water-deficit and heat stress treatments in the year 2017–2018. The water-deficit stress, heat stress, and combined water-deficit and heat stress environments increased SRE by 28%–80%, 13%–61%, and 60%–95%, respectively, over the non-stressed environment (*p* < 0.05). The BLUE model explained that the genotype × environment interaction was highly significant on SRM (*p* < 0.001) (Table 2). As compared to the non-defoliated plants, the mean SRM values were higher in the defoliated plants, and the incremental change in SRM over the non-defoliated plants was found in the order non-stress (59%) heat stress (10%), water-deficit stress (7%), and combined water-deficit and heat stress (4%) environments (Figure 3E).

**FIGURE 4:**
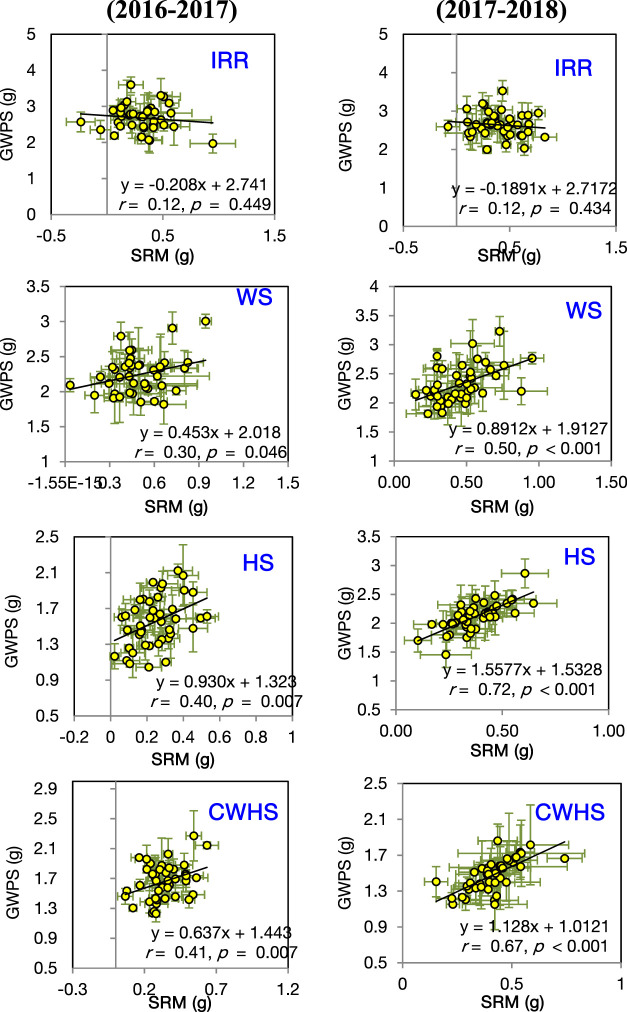
Linear relationship between stem reserve mobilization (SRM) and grain weight spike^−1^ (GWPS) in the year 2016–2017 and 2017–2018 under different crop growing environments. IRR, irrigated (non-stress); WS, water-deficit stress; HS, heat stress; and CWHS, combined water-deficit and heat stress. The vertical and horizontal error bars represents the corresponding standard error of means.

**FIGURE 5:**
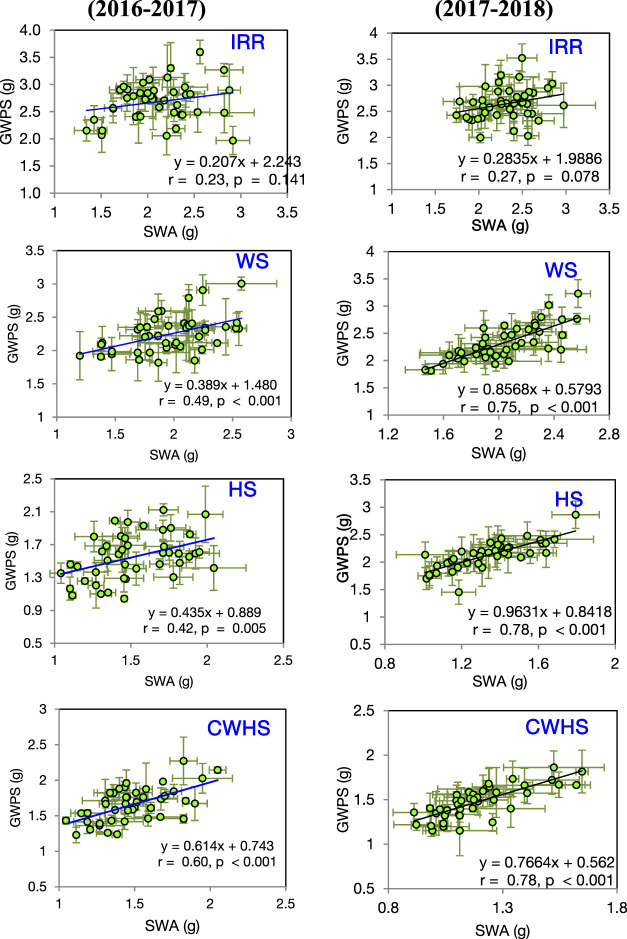
Linear correlations between single stem (main stem) weight at 12 days after anthesis (SWA) and grain weight spike^−1^ (GWPS) under different crop growing environments in the year 2016–2017 and 2017–2018. IRR, irrigated (non-stress); WS, water-deficit stress; HS, heat stress; and CWHS, combined water-deficit and heat stress. The vertical and horizontal error bars represents the corresponding standard error of means.

**FIGURE 6:**
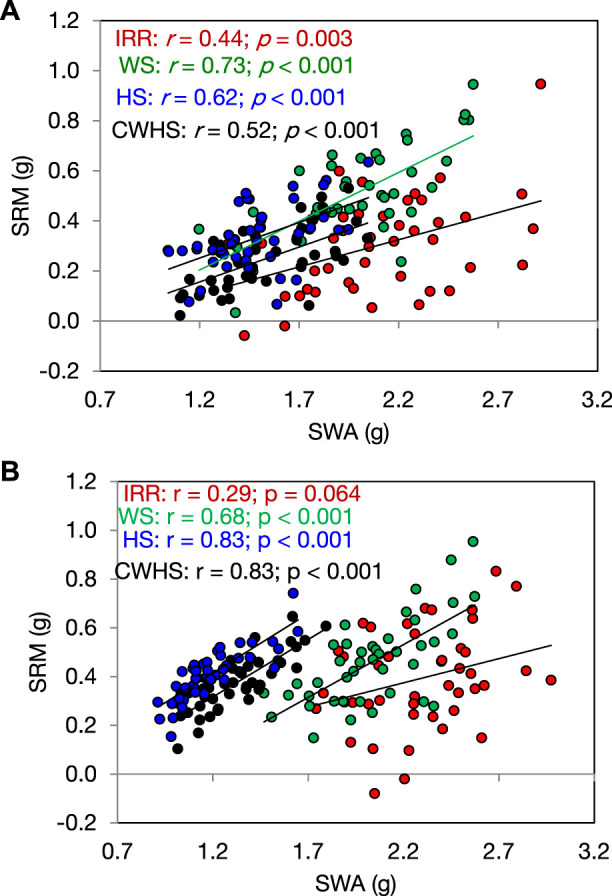
Relationship between stem weight [main stem weight at 12 days after anthesis (SWA)] and stem reserve mobilization (SRM) under different crop growing environments in the year 2016–2017 **(A)** and 2017–2018 **(B)**. IRR, irrigated (non-stress); WS, water-deficit stress; HS, heat stress; and CWHS, combined water-deficit and heat stress.

**FIGURE 7:**
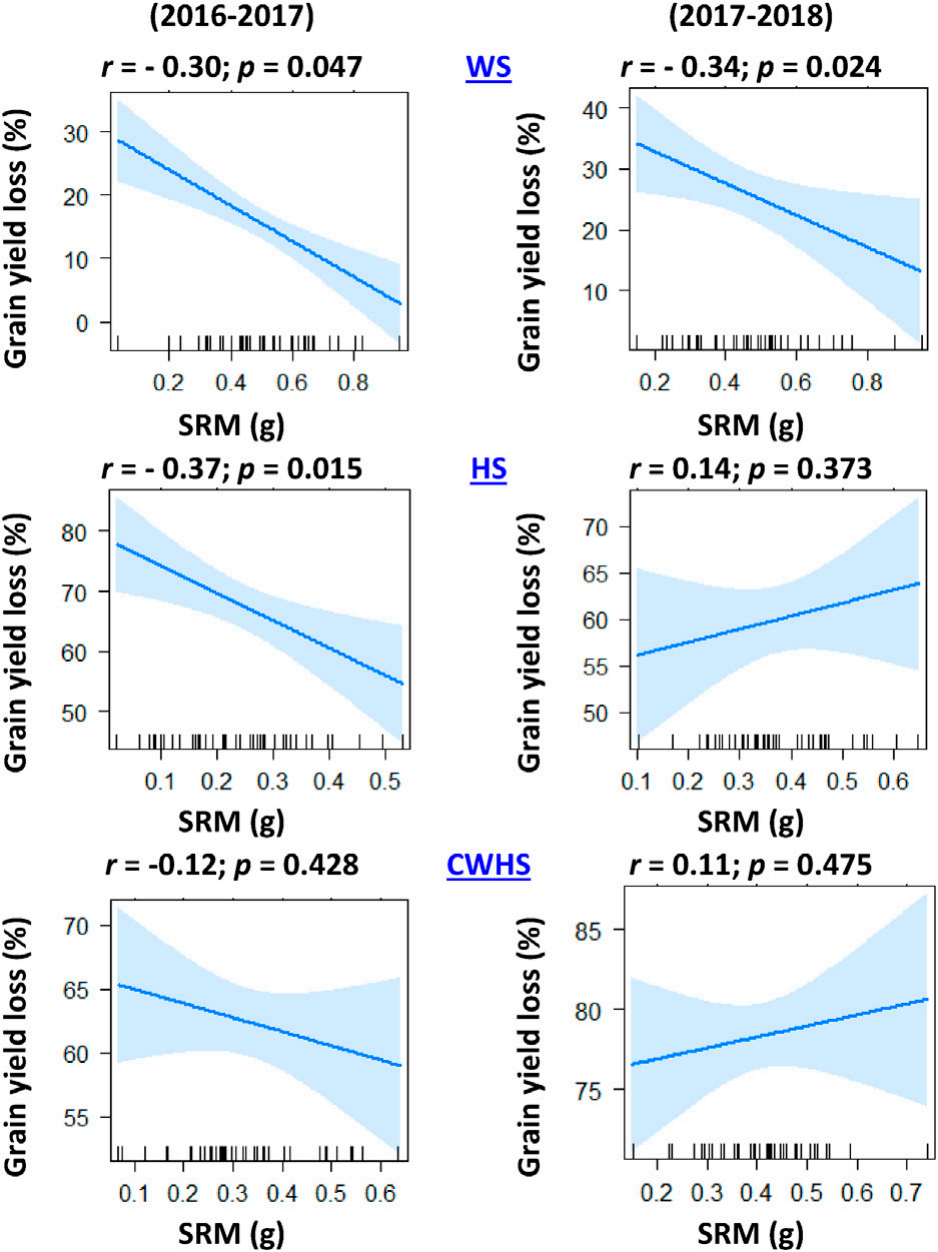
Linear regression trend line explaining the association between grain yield loss (loss as compared to irrigated non-stress treatment) and stem reserve mobilization (SRM) under different stressed conditions in the year 2016–2017 and 2017–2018. IRR, irrigated (non-stress); WS, water-deficit stress; HS, heat stress; and CWHS, combined water-deficit and heat stress.

In both years, SRM and SRE had a non-significant correlation with grain weight spike^−1^ in the non-stress environment (Figure 4). Under the stress environments (water-deficit stress, heat stress, and combined water-deficit and heat stress), SRM exhibited a significant positive correlation with grain weight spike^−1^ (*p* < 0.05). The correlation coefficient (*r*) between SRM and grain weight spike^−1^ was the highest under the heat stress environment. Stem weight (at 12 DAA) and grain weight spike^−1^ were positively correlated under the stress environments, but not under the non-stress environment (Figure 5). Meanwhile, the significant correlations between stem weight (anthesis) and SRM were observed under all stress environments, and the results were consistent in both years (Figure 6). Under the water-deficit stress conditions, with an increase in SRM there was a significant reduction in the yield loss (yield loss calculated comparing with non-stress environment) (Figure 7). Under the heat stress environment, the SRM trait exhibited a negative relation with the yield loss in the year 2016–2017 (*p* < 0.05), but not in 2017–2018. The relationship between yield loss and SRM under the combined water-deficit and heat stress environments was non-significant in both years.

### Genotypic variability and genotype × environment interaction

**FIGURE 8:**
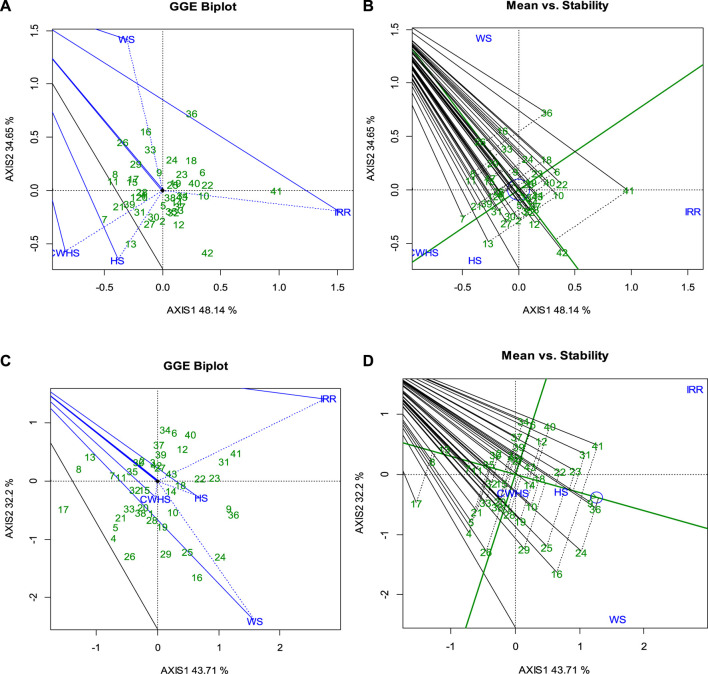
GGEBiplot presentation to explain the genotype × environment interactions for the trait SRM in the year 2016–2017 **(A)** and 2017–2018 **(C)**; Mean stability of wheat genotypes for SRM trait under different environments in the year 2016–2017 **(B)** and 2017–2018 **(D)**. IRR, irrigated (non-stress); WS, water-deficit stress; HS, heat stress; and CWHS, combined water-deficit and heat stress.

Noticeable genotypic variations were observed for the SRM trait. Particularly, under the water-deficit stress environment, SRM varied between 0.15 and 0.95 g (2016–2017) and 0.03–0.95 g (2017–2018); SRE values ranged between and 3%–37% and 9%–37%. Under the non-stress environment, genotypes PBW 343, WH 542, GCP 30, and HD 2864 consistently had a higher SRM (>0.5 g stem^–1^) and SRE (>30%) over the remaining genotypes; under the water-deficit stress condition, the genotypes HI 8627, C 306, WH 542, HD 4728, RAJ 3765, and GCP 30 were found to have a higher SRM (>0.6 g stem^–1^) and SRE (>30%) over the remaining genotypes. Considering the parameter yield loss (<15%), SRM (>0.4 g stem^–1^) and SRE (>25%) across the year, the genotypes C 306, GCP 29, and GCP 1 were found to be the best genotypes under the water-deficit stress environment. GGEbiplot analysis revealed that there was a differential sensitivity of the selected genotypes for the trait SRM with a strong G × E interaction (Figure 8). As shown in the mean stability graph, notable variation in the SRM trait was apparent within the experimental year (Figure 8).

### Other important correlations

Stem-specific weight exhibited positive associations with SRE and SRM across the environment (Table 4). A non-significant association was observed between SRM and grain weight spike^−1^ in defoliated plants; however, stem-specific weight had a strong positive correlation with grain weight spike^−1^ but not with SRM. Similar to the non-defoliated plant, stem weight at anthesis had a positive association with SRM in defoliated plants across the environment (Supplementary Table S2).

**TABLE 4:** Pearson correlation coefficients (*r*) of stem reserve mobilization parameters with growth and yield parameters under different crop growing environments.

Year	Environment	Parameter	SWM	STHA	SSWA	GDD	GWP	HI	[GW/SW]
2016–2017	Irrigated (non-stress)	SRM	−0.09	−0.10	0.69***	−0.27	0.97***	0.11	0.02
		SRE	−0.35*	−0.27	0.54***	−0.18	0.90***	0.30*	0.26
	Water-deficit stress	SRM	0.28	0.20	0.69***	−0.19	0.93***	−0.25	−0.12
		SRE	−0.16	−0.04	0.54***	−0.22	0.90***	−0.04	0.22
	Heat stress	SRM	0.21	0.12	0.65***	0.10	0.92***	0.41**	0.18
		SRE	−0.12	−0.11	0.50***	0.09	0.91***	0.48**	0.38*
	Combined water-deficit and heat stress	SRM	−0.04	−0.06	0.67***	−0.01	0.91***	0.29	0.33*
		SRE	−0.44**	−0.33*	0.48**	−0.18	0.92***	0.35*	0.56***
2017–2018	Irrigated (non-stress)	SRM	−0.38*	−0.24	0.58***	−0.20	0.96***	0.09	0.30*
		SRE	−0.61***	−0.36*	0.50***	−0.20	0.94***	0.12	0.48**
	Water-deficit stress	SRM	0.05	−0.10	0.39**	−0.26	0.93***	−0.36*	0.50***
		SRE	−0.25	−0.26	0.34*	−0.30	0.95***	−0.26	0.60***
	Heat stress	SRM	0.41**	−0.19	0.36*	−0.01	0.93***	−0.08	0.26
		SRE	−0.05	−0.43**	0.24	−0.02	0.90***	−0.01	0.59***
	Combined water-deficit and heat stress	SRM	0.40**	−0.02	0.67***	−0.08	0.88***	0.28	0.25
		SRE	−0.21	−0.07	0.49***	−0.08	0.85***	0.30*	0.59***

*SRM,* stem reserve mobilization; *SRE,* stem reserve mobilization efficiency; *SWM,* single stem weight at maturity; *STHA,* stem height at anthesis; *SSWA,* stem-specific weight at anthesis; *LDR,* leaf senescence rate; *LSD,* leaf senescence duration; *GDD,* cumulative growing degree days; *GWP,* grain weight percentage; *HI,* harvest index; *GW*/*SW,* grain/stem weight ratio. The correlation (*r*) values highlighted with green, yellow, and pink colors are significant at *p* < 0.001, *p* < 0.01, and *p* < 0.05, respectively.

## Discussion

The soil moisture data indicate that the crop experienced severe soil moisture-deficit stress (<10% w/w) during the post-anthesis stages in both water-deficit stress and combined water-deficit and heat stress treatments. Furthermore, during the reproductive stages, the ambient temperatures in the heat stress and combined water-deficit and heat stress treatments were significantly higher (23.9–29.7°C) than the optimal temperature for wheat (12–22°C) (Dwivedi et al., 2017). Thus, the results confirm that the terminal drought and heat stress conditions were effectively induced in this study.

Our findings demonstrate that water-deficit stress can enhance SRM and SRE in wheat. This aligns with previous studies that have also reported increased SRM in wheat under water-deficit stress when compared to well-irrigated non-stress conditions (Asseng and Van Herwaarden, 2003; Ruuska et al., 2006; Thapa et al., 2022). Under both drought and high-temperature environments, post-anthesis photosynthesis is affected, resulting in an increased reliance on pre-anthesis soluble carbohydrate reserves, especially from the stem (Ruuska et al., 2006; Tatar et al., 2016). Meanwhile, drought conditions have been shown to accelerate the process of reserve mobilization to grain (∼10 days earlier) when compared to well-irrigated conditions (Ehdaie et al., 2008). In our study, the wider genotypic variations observed in both SRM and SRE under water-stress environments, as compared to other environment, suggest that the inherent capacity of plants to mobilize reserves is heavily influenced by crop-growing environments. Conversely, heat stress conditions resulted in reduced SRM, which may be attributed to reduced stem biomass at anthesis and significant reduction in the grain-filling duration (Ovenden et al., 2017; Islam et al., 2021). Our results indicate that wheat is more sensitive to terminal heat stress in a subtropical climate than to drought stress. Furthermore, the negative effects of heat stress on sink capacity may also be an important contributing factor to the lower SRM under heat stress environment.

The correlation analysis reveals that stem-assimilate reserve capacity (or stem biomass), stem-specific weight, and grain-filling duration are influential factors for SRM in wheat. Notably, a significant correlation between SRM and main stem grain weight was only observed under stress treatments. This suggests that the significance of the SRM trait for grain development is limited to stress conditions, and despite an increased scale of SRM under non-stress conditions, its relative contribution toward grain development is minimal. In the absence of abiotic stress factors, normal plant functions facilitate higher current photosynthate assimilation during the post-anthesis period, largely contributing toward developing grain; subsequently, the contribution of SRM is reduced. All such individual growth factors (fertilization, crop rotation, and plant protection measures) that directly or indirectly influence plant growth and development determine SRM. Therefore, the SRM trait may serve as a conditional functional trait, and its relative significance for grain development is dependent on several factors, such as the nature and intensity of stress factors, stem reserve capacity, phenological stability (especially the grain-filling duration), and sink capacity.

The significant negative correlation between SRM and grain yield loss (compared to the non-stress condition) highlights the potential of SRM as a trait for improving yield sustainability, particularly under water-deficit stress conditions. However, the contribution of SRM toward yield sustainability under heat stress conditions remains uncertain from the study results. Despite the strong positive relationship between SRM and grain weight spike^−1^ under heat stress environments (*i.e.*, heat stress and combined water-deficit and heat stress), the influence of SRM on yield may have been impacted by the oversized influence of heat stress on sink inefficiencies and effective tiller production (data not presented). Previous studies in the IGP regions have reported inefficiencies at the sink development in late-sown crops, which are attributed to reduced functionality of reproductive organs such as pollen viability and embryo abortion (Bheemanahalli et al., 2019; Chowdhury et al., 2020). The imbalance in source-sink relations, particularly the reduction in the sink, may be a reason for the noticeable yield loss under heat stress environment. Studies have reasoned that an optimal balance in source and sink has a greater role in the mobilization of stored carbohydrates (Yu et al., 2015; Sehgal et al., 2018; Rivera-Amado et al., 2020). It is possible that under heat stress and combined water-deficit and heat stress environments, the impact of the environment on yield was much greater than that of the genotype, which might have confounded the correlation results.

The results of the defoliation study suggest that limiting current photosynthate assimilation during post-anthesis stages may trigger the reserve mobilization process (Liu et al., 2018); however, according to results, an increase in SRM does not essentially translate to grain development. Defoliation of wheat plant after anthesis may have caused major inefficiencies in physiological functions that have a direct impact on stem reserves mobilization efficiency. Notably, the maximum reduction in grain weight spike^−1^ in defoliated plants was 36%, with grain weight percentages ranging between 24% and 36%, indicating that active photosynthesis by the stem, awn, and other photosynthetically active plant parts may have contributed to grain development. Saeidi et al. (2012) and Zhang et al. (2020) suggested that spike photosynthesis has a greater role in yield formation under stressed conditions than carbohydrate remobilization and leaf photosynthesis. Therefore, the actual values of SRM may be higher than measured, and the observed SRM may be somewhat underestimated.

The study showed that the traits SRM and SRE exhibited wider variations among genotypes under all environments, with higher variations under water-deficit stress conditions. This variability could be attributed to differences in stem-soluble carbohydrate concentrations among the genotypes (Ehdaie et al., 2006; Ruuska et al., 2006; Liu et al., 2020; Vosoghi Rad et al., 2022). Soluble carbohydrates in the stem have been found to mobilize into the grain during the grain-filling period, and stem biomass after anthesis is directly related to the total water-soluble carbohydrate in the stem under water-deficit conditions (Saint Pierre et al., 2010). According to Ruuska et al. (2006), the variation in total water-soluble carbohydrate was attributed mainly to variations in the fructan component, with the other major soluble carbohydrates, sucrose, and hexose varying less. Therefore, the study suggests that the ability to accumulate and remobilize stem reserves is an important trait for improving grain filling in wheat under water-limited environments. The SRM trait could be particularly useful for developing drought-tolerant cultivars; however, its significance under tropical heat stress conditions may be limited.

Previous research showed that genotypes with high potential for utilizing stem reserves for developing grains under stress conditions may be associated with accelerated leaf senescence dynamics (Fokar et al., 1998; Islam et al., 2021; Salem et al., 2021). Therefore, investigating the relationships of SRM with grain-filling duration, leaf senescence dynamics, and source and sink components under different environmental conditions could provide valuable insights. Additionally, molecular studies could better characterize the trait and to understand the mechanism that could help in developing wheat varieties with improved SRM efficiency using appropriate bio-engineering approaches. The heritability estimates of 49% and 56% for the traits SRM and SRE, respectively, suggest that a significant proportion of the observed genotypic variations were due to the genotypic effects (Table 2). Thus, developing and deploying wheat varieties using the SRM trait could be a promising approach to address the challenges of abiotic stresses in tropical agro-regions. The GGEbiplot analysis was found effective in categorizing wheat genotypes based on the SRM trait and representing genotype × environment interactions. According to GGEbiplot analysis, the genotype-by-environment interaction was notably higher (represented by the Axis 2 component) suggesting that the genotypic expression of SRM is highly environment specific. Therefore, unique genotypes should be selected, and distinct selection strategies may be employed for different environments. The genotype(s) with higher and stable SRM capacity across different environments would be ideal to select as valuable genetic resources for the future breeding program. According to the results, genotype 41 (HD 2864) and genotypes 10 (GCP 6) could serve as potential genetic resources for SRM as a trait, as these genotypes exhibited higher SRM in both years.

Overall, stem reserve mobilization is a significant trait for improving the tolerance of plants to water-deficit stress conditions, which is important for the sustainability of agriculture and food security in the face of climate change and other environmental challenges. The impact of weather variability and climate change on wheat yields, therefore, remains central to food security concerns and directly affects the livelihood of small-scale farmers who control the majority of the landholdings in India and produce 41% of India’s food grains (Zaveri, and Lobell, 2019). Furthermore, wheat productivity in the IGP region is largely dependent on irrigation, and the evident depletion of groundwater resources poses a major threat to wheat production in the region. Recent evidence points to irrigation as one of the key factors in explaining wheat yield gaps across the western and eastern parts of the Indo-Gangetic Plain (Jain et al., 2017). In some parts, the IGP regions are already facing moderate-to-severe water scarcity. In that consideration, enhancing plant SRM capacity could be an economic and realistic approach. Therefore, improving stem reserve mobilization in crops would be an important goal for plant breeders and geneticists, as it can lead to more resilient and productive plants under water-limited environments.

## Conclusion

The study concluded that the SRM trait has an increased significance in sustaining wheat yield particularly under stressful tropical environments (water-deficit stress in particular). Water-deficit stress had enhanced SRM over the non-stress environment. Results suggested SRM and stem biomass (or stem reserve capacity) positively influenced the grain development (measured as grain weight spike^−1^) under stressful environments, but not under the non-stress (timely sown well irrigated) condition. However, the SRM-mediated grain yield (yield area^−1^) protection was only evident under the water-deficit stress but not under the heat stress environment, demonstrating the significance of the SRM trait for yield advantage is not certain under heat stress environments, which is possibly attributed to sink inefficiencies and physiological dysfunctions. The defoliation study revealed that in the absence of post-anthesis photosynthesis by leaves, the contribution of SRM toward grain development was substantially increased (36%), being higher under the non-stress environment over the stress environment. The wider genotypic variations in the SRM trait and the yield protection attributed to SRM under water-deficit (drought) stress validated the approach, which needs to be integrated in the wheat-breeding program.

## Data Availability

The raw data supporting the conclusion of this article will be made available by the authors, without undue reservation.
