# Supplementary material for: Genotypic capacity of post-anthesis stem reserve mobilization in wheat for yield sustainability under drought and heat stress in the subtropical region
Source: Front Genet. 2023 Jun 20;14:1180941. doi: 10.3389/fgene.2023.1180941 (PMC10318140; doi:10.3389/fgene.2023.1180941)
Supplement: Supplementary file 1 [file DataSheet1.docx]

**Supplementary Table 1** Pedigree and ploidy level of the selected wheat genotypes

| **S. N** | **Genotype** | **Pedigree** | **Ploidy** |
| --- | --- | --- | --- |
| 1 | HD 2932 | KAUZ/STAR//HD 2643 | 6x |
| 2 | DHARWAR DRY | Selection from local line | 6x |
| 3 | GCP 36 | DBW43/HI1500 | 6x |
| 4 | WL 711 | S308/CHR/KAL | 6x |
| 5 | HD 2987 | HI 1011/HD 2348//MENDOS//IWP 72/DL 153-2 | 6x |
| 6 | PBW 343 | ND/VG9144//KAL/BB/3/YCO"S'/4/VEE#S "S" | 6x |
| 7 | PBW 550 | WH 594/RAJ 3858//W 485 | 6x |
| 8 | HINDI 62 | PBW-226/HW-1042//HD-2285 | 6x |
| 9 | HI 8627 | HD 4672/ PDW 233 | 4x |
| 10 | GCP 6 | DBW43/HI1500 | 6x |
| 11 | GW 322 | KUKRI/RAC875 | 6x |
| 12 | HD 2733 | ATTILA /3/TUI /CARC //CHEN / CHTO /4/ATTILA | 6x |
| 13 | WR 544 | KALYANSONA/HD 1999//HD 2204/DW 38 | 6x |
| 14 | HI 1544 | HINDI 62/BOBWHITE/CPAN2099 | 6x |
| 15 | GCP 25 | DBW43/HI1500 | 6x |
| 16 | C 306 | RGN/CSK3//2*C5 91/3/C217/N14 //C281 | 6x |
| 17 | HD 2967 | ALD/COC//URES/HD216 0M/HD2278 | 6x |
| 18 | GCP 29 | DBW43/HI1500 | 6x |
| 19 | HD 3086 | DBW14/HD2733//HUW468 | 6x |
| 20 | CHIRYA 7 | CYMMIT line | 6x |
| 21 | NP 4 | SEL.LINE OF LOCAL MUNDIA | 6x |
| 22 | WH 542 | JUP /BJY”S”//URES | 6x |
| 23 | HD 2687 | CPAN 2009/ HD 2329 | 6x |
| 24 | GCP 2 | DBW43/HI1500 | 6x |
| 25 | HD 4728 | ALTAR84/STINT/SILVER 453/ SOMAT 3.1/4/GREEN14/YAV10 /AUK | 4x |
| 26 | RAJ 3765 | HD 2402/VL639 | 6x |
| 27 | KUNDAN | TANORI 71/ NP 890 | 6x |
| 28 | GCP 1 | DBW43/HI1500 | 6x |
| 29 | GCP 23 | DBW43/HI1500 | 6x |
| 30 | Kalyansona | PJ'S'/GB 55 | 6x |
| 31 | DBW 43 | Babax/LR-42//Babax*2/3/Vivits | 6x |
| 32 | CHIRYA 3 | CYMMIT line | 6x |
| 33 | HD 3059 | KAUZ//ALTAR84/AOS/3/MILAN/KAUZ/4/HUITES | 6x |
| 34 | HD 2329 | SLSIB/NP852/4/PJSIB/P14//KT54B/K65/5/SKA/6/UP262 | 6x |
| 35 | HD 2851 | CPAN 3004/WR 426//HW 2007 | 6x |
| 36 | GCP 30 | DBW43/HI1500 | 6x |
| 37 | Westonia | CO-1190-203/84-W-127-501 | 6x |
| 38 | WH 730 | CPAN2092/Improved Lok1 | 6x |
| 39 | HD 2985 | PBW 343/ PASTOR | 6x |
| 40 | GCP 16 | DBW43/HI1500 | 6x |
| 41 | HD 2864 | DL 509-2/ DL 377- 8 | 6x |
| 42 | HALNA | HD 1982/K816 | 6x |
| 43 | BABAX | BOBWHITE/NACOZARI-76//VEERY/3/BLUEJAY/COCORAQUE-75 | 6x |

**The genotypes selection criterion (seven groups)**

(1) RILs: 9 + 2 parents

| RILs (HI 1500 × DBW 43) | | | |
| --- | --- | --- | --- |
| SN | Genotype | SN | Genotype |
| 01 | GCP P 1 | 07 | GCP P 29 |
| 02 | GCP P 2 | 08 | GCP P 30 |
| 03 | GCP P 15 | 09 | GCP P 36 |
| 04 | GCP P 16 | 10 | GCP P 49 |
| 05 | GCP P 23 | 11 | GCP P 6 |
| 06 | GCP P 25 |  |  |

(2) Contrasting heat stress genotypes: 6 + 6

| Contrasting heat stress genotypes | | | |
| --- | --- | --- | --- |
| SN | Heat tolerant genotypes | SN | Heat susceptible genotypes |
| 01 | WH 730 | 01 | HD 2733 |
| 02 | Hindi 62 | 02 | GW 322 |
| 03 | RAJ 3765 | 03 | PBW 343 |
| 04 | HALNA | 04 | PBW 550 |
| 05 | HD 2985 | 05 | HD 2687 |
| 06 | CHIRIYA 7 | 06 | WH 542 |

(3) Contrasting drought stress genotypes: 7 + 7

| Contrasting drought stress genotypes | | | |
| --- | --- | --- | --- |
| S/N | Drought tolerant genotype | S/N | Drought susceptible genotype |
| 01 | C 306** | 01 | HD 2329* |
| 02 | Dharwad dry | 02 | WL 711 |
| 03 | DBW 43 | 03 | HD 2733 |
| 04 | KUNDAN | 04 | GW 322** |
| 05 | HD 2932* | 05 | PBW 550** |
| 06 | Babax | 06 | HD 2687*** |
| 07 | HD 2987** | 07 | PBW 343** |

(4) Contrasting height genotypes: 4 + 5

| Contrasting height genotypes | | | |
| --- | --- | --- | --- |
| S/N | Tall | S/N | Dwarf |
| 01 | C 306** | 01 | HD 2851 |
| 02 | Dharwad dry | 02 | HD 2864 |
| 03 | KUNDAN | 03 | HD 2987** |
| 04 | NP 4 | 04 | HD 3086 |
|  |  | 05 | HD 2687*** |

(5) Genotypes with duration of maturity: 4 + 4

| Genotypes with duration of maturity | | | |
| --- | --- | --- | --- |
| S/N | Early maturing genotypes | S/N | Late maturing genotypes |
| 01 | HALNA* | 01 | RAJ 3765* |
| 02 | HD 2987** | 02 | HD 2932* |
| 03 | WH 730* | 03 | HD 3059 |
| 04 | WR 544* | 04 | HD 2967 |

(6) Current best standard genotypes: best-adapted varieties: 6

| Current best standard genotypes | | | |
| --- | --- | --- | --- |
| S/N | Genotypes | S/N | Genotypes |
| 01 | HD 2967 | 04 | HD 2733* |
| 02 | HD 3086 | 05 | PBW 550** |
| 03 | GW 322** | 06 | HI 1544 |

(7) Popular ruling varieties of last century: 10

| Popular ruling varieties of last century | | | |
| --- | --- | --- | --- |
| S/N | Genotypes | S/N | Genotypes |
| 01 | NP 4 (1900s) | 06 | PBW 343 (1995)** |
| 02 | C 306 (1965)** | 07 | HD 2687 (1999)*** |
| 03 | KALYANSONA (1970s) | 08 | GW 322 (2006)** |
| 04 | HD 2864 (1970s) | 09 | HD 2967 (2011) |
| 05 | HD 2329 (1985)* | 10 | HD 3086 (2014)*** |

**Supplementary Fig. 1** Weather conditions [daily rainfall (mm), evaporation rate (mm), minimum and maximum temperatures (^o^C)] during the crop season of 2016-2017 and 2017-2018

**Supplementary Fig. 2** Weather condition [daily rainfall (mm), pan evaporation rate (mm), minimum and maximum temperatures (^o^C)] during the crop season of 2016-2017 and 2017-2018 and temporal distribution of flowering time [green line (first quartile), pink line (second quartile), blue line (third quartile), red line (fourth quartile)] under different crop growing environments. *IRR*, irrigated (non-stress), *WS*, water-deficit stress, *HS*, heat stress, and *CWHS*, combined water-deficit and heat stress. Each data point represents a single genotype.

**Supplementary Table 2** Linear relationship (Pearson correlation coefficient) of stem reserve mobilization parameters with growth and yield parameters of defoliated wheat crop under different crop growing environments (defoliation study, 2016-2017)

| Parameter | | SRE | SRM | GWPS | GWP | SWA | SWM | SHM |
| --- | --- | --- | --- | --- | --- | --- | --- | --- |
| Irrigated  (non-stress) | SRE |  |  |  |  |  |  |  |
|  | SRM | 0.88 |  |  |  |  |  |  |
|  | GWPS | -0.39 | -0.10 |  |  |  |  |  |
|  | GWP | 0.93 | 0.94 | -0.41 |  |  |  |  |
|  | SWA | 0.05 | 0.49 | 0.54 | 0.27 |  |  |  |
|  | SWM | -0.50 | -0.06 | 0.68 | -0.27 | 0.84 |  |  |
|  | SHM | -0.19 | 0.09 | 0.19 | 0.02 | 0.59 | 0.62 |  |
|  | SSW | -0.53 | -0.21 | 0.86 | -0.48 | 0.56 | 0.77 | 0.15 |
| Water-deficit stress | SRE |  |  |  |  |  |  |  |
|  | SRM | 0.88 |  |  |  |  |  |  |
|  | GWPS | -0.24 | 0.09 |  |  |  |  |  |
|  | GWP | 0.92 | 0.87 | -0.38 |  |  |  |  |
|  | SWA | 0.21 | 0.63 | 0.53 | 0.34 |  |  |  |
|  | SWM | -0.38 | 0.08 | 0.62 | -0.20 | 0.82 |  |  |
|  | SHM | -0.17 | 0.08 | 0.20 | -0.02 | 0.51 | 0.60 |  |
|  | SSW | -0.29 | -0.05 | 0.69 | -0.36 | 0.35 | 0.49 | -0.22 |
| Heat stress | SRE |  |  |  |  |  |  |  |
|  | SRM | 0.93 |  |  |  |  |  |  |
|  | GWPS | 0.03 | 0.15 |  |  |  |  |  |
|  | GWP | 0.92 | 0.94 | -0.15 |  |  |  |  |
|  | SWA | 0.31 | 0.61 | 0.44 | 0.47 |  |  |  |
|  | SWM | -0.19 | 0.13 | 0.45 | -0.01 | 0.87 |  |  |
|  | SHM | -0.05 | 0.15 | 0.14 | 0.14 | 0.59 | 0.63 |  |
|  | SSW | -0.27 | -0.10 | 0.58 | -0.26 | 0.43 | 0.60 | -0.05 |
| Combined water -deficit and heat stress | SRE |  |  |  |  |  |  |  |
|  | SRM | 0.87 |  |  |  |  |  |  |
|  | GWPS | -0.34 | -0.07 |  |  |  |  |  |
|  | GWP | 0.90 | 0.89 | -0.50 |  |  |  |  |
|  | SWA | 0.04 | 0.51 | 0.47 | 0.23 |  |  |  |
|  | SWM | -0.46 | 0.01 | 0.59 | -0.24 | 0.87 |  |  |
|  | SHM | -0.26 | 0.02 | 0.08 | 0.00 | 0.57 | 0.65 |  |
|  | SSW | -0.44 | -0.12 | 0.80 | -0.47 | 0.52 | 0.67 | 0.05 |

*SRM,* stem reserve mobilization; *SRE,* stem reserve mobilization efficiency; *GWPS*, grain weight spike^-1^; *GWP*, grain weight percentage; *SWA*, stem weight at anthesis; *SWM,* stem weight at maturity; *SHM,* stem height at maturity. The correlation (*r*) values highlighted with green, yellow, and pink colour are significant at *p* < 0.001, *p* < 0.01, and *p* < 0.05, respectively.
